# Evaluation of strain values of critical anatomic regions for two different pterygomaxillary approaches in Le Fort I osteotomy: An experimental study

**DOI:** 10.4317/medoral.21663

**Published:** 2017-04-08

**Authors:** Yusuf-Bugra Ozdemir, Dogan Dolanmaz, Alparslan Esen, Hakan Terzioglu, Haci Saglam

**Affiliations:** 1 DDS PhD (Research Assistant), Selcuk Universtiy, Faculty of Dentistry, Department of Oral and Maxillofacial Surgery, Konya/Turkey; 2 DDS PhD (Professor), Selcuk Universtiy, Faculty of Dentistry, Department of Oral and Maxillofacial Surgery, Konya/Turkey; 3 DDS PhD (Assistant Professor), Necmettin Erbakan Universtiy, Faculty of Dentistry, Department of Oral and Maxillofacial Surgery, Konya/Turkey; 4 PhD (Lecturer), Selcuk University, Vocational School of Technical Sciences, Division of Electricity and Energy, Konya/Turkey; 5 PhD (Professor), Selcuk Universtiy, Faculty of Technology, Department of Mechanical Engineering Konya/Turkey

## Abstract

**Background:**

The purpose of this experimental study was to measure stresses both on the pterygoid plates and the skull base following two different pterygomaxillary approaches in Le Fort I osteotomy.

**Material and Methods:**

The prepared skull models were randomly divided into 2 groups of 7. In the first group (A), the pterygomaxillary area was left intact. In the second group (B), pterygomaxillary separation was performed with a fine bur. The stresses were measured by using strain gauges. These strain gauges were attached to 6 different anatomical sites. The skull models were mounted on a servo-hydraulic testing unit. Each model was then subjected to a continuous linear tension until a plastic deformation was seen.

**Results:**

The statistical analyses showed that there were no significant differences (*p* >.05) between the 2 groups regarding the strain values. Moreover, no statistical differences (*p* >.05) were found between the two groups in terms of maximum applied forces.

**Conclusions:**

Considering the clinical conditions, the present study shows that when Le Fort I osteotomy performed without pterygomaxillary separation, there is no significant stress on the skull base during the downfracture. Moreover, it is considered that there is no need for an excessive force applied to perform downfracture in Le Fort osteotomies without pterygomaxillary separation.

** Key words:**Le Fort I, osteotomy, strain, base of skull, pterygoid process.

## Introduction

Le Fort I osteotomy is a widely applied technique for correction of maxillofacial deformities. Although low complication rates have been reported (1-6), these complications have range from mild to severe and there is no consensus about the reasons of the complications following Le Fort I osteotomy (3,7). Many researchers have defended that the complications derive mainly from pterygomaxillary osteotomy (2,8,9). Therefore, several experimental studies have been performed to explain the mechanism of this complications (9-11). These studies have focused on only pterygoid plates in general. To our knowledge, there is only one study about the evaluation of strain distribution on pterygomaxillary area after Le Fort I osteotomy (12). However; there is no study about the evaluation of the strains occurring both on the pterygoid plates and base of the skull during the downward mobilization of the maxilla.

It is considered that the neurological complications may depend on more than one reason. These reasons which may cause nerve damage can be mentioned as the indirect compression or traction affecting the skull base, direct trauma during pterygomaxillary osteotomy and difficult downfracture (3). Therefore, in the present study the purpose was to measure stresses both on the pterygoid plates and the skull base following two different pterygomaxillary approaches in Le Fort I osteotomy.

## Materials and Methods

The study protocol was approved by the Ethics Committee of the University. We used slices from a 3-dimensional computed tomogram (CT) of an adult patient with full dentition to make 14 composite models similar to the hardness of the bone structure. Standard Le fort 1 osteotomy was carried out on all skull models. All the cuts were standardized with reference to certain anatomical landmarks such as the medial and distal orbital wall, the zygomatic buttress, and the teeth. We divided the 14 skull models into two groups. In the first group (A), we did not perform any osteotomy in the pterygomaxillary junction. In the second group (B), we performed pterygomaxillary separation with a fine bur.

**Figure 1:**
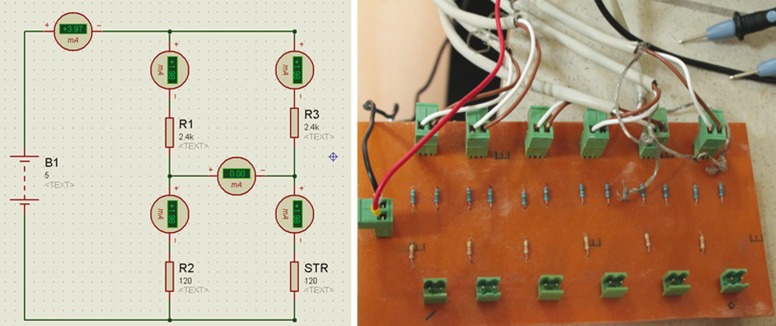
Diagram of the designed quarter WB for each strain gauge and the picture of WB. As shown in the diagram, the circuit is in balance since there is no current in the bridge output.

In the present study, the strains were measured by using 120 Ω, GFLA-3-50-2L long strain gauges (Tokyo Sokki Kenkyujo Co. Ltd., Japan). These strain gauges have a feature that resistance variation when the force exerted on them. Therefore, they should be used with a Wheatstone Bridge (WB). WB is an electrical circuit used to measure an electrical resistance. The primary benefit of a WB is its ability to provide extremely accurate measurements. Each strain gauge was connected to the quarter WB and a total of 6 quarter WB was designed in the present study (Fig. 1). Due to the signals obtained from the WB were low, we used an amplifier circuit (ADAM 3016 Isolated Strain Gauge Input Module, Advantech, California, USA) that it’s inputs and outputs can be set by switches to amplify the signals. In this configuration, the input signal was set to 20 mA whereas the output signal was set to ± 10 V because of the direction of force that can be positive or negative.

The strain values produced from the amplifier circuit were transferred by a data processing card (PCI 1710HG-Advantech, California, USA) to the computer and the data were recorded through a software program at 10 msec intervals (Mat lab-Simulink, Math works, Massachusetts, USA) during the test. The strain values were obtained in volts at the end of the test. Hence, a second experiment was performed to detect equivalents of these values as newton. We applied force with a dynamometer on a flexible plate attached to the strain gauge. Read values of force on the dynamometer and strain values were calibrated based on the linear relationship of the force and strain. As a result, 1mV is considered to be 0.5 N.

**Figure 2:**
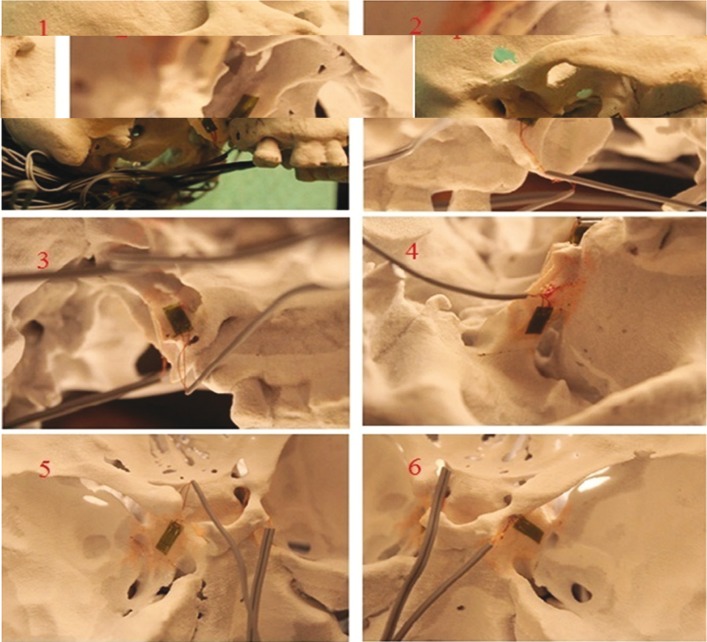
The strain gauges were attached on the 6 different anatomical region of the skull. These regions; 1: The lateral surface of right lateral pterygoid plate, 2: The medial surface of right medial pterygoid plate, 3: The medial surface of left medial pterygoid plate, 4: The lateral surface of left lateral pterygoid plate, 5: The lower surface of the entrance of right canalis opticus, 6: The lower surface of the entrance of left canalis opticus.

**Figure 3:**
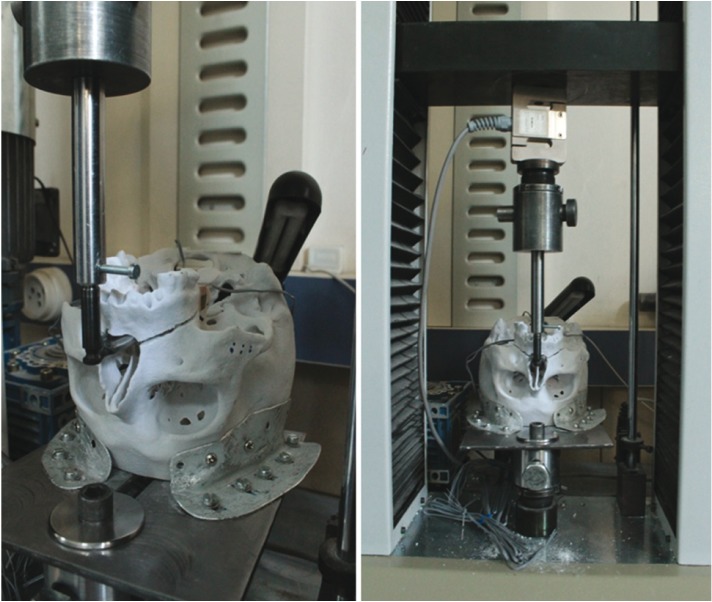
The stereolithographic skull models mounted on a fixation apparatus with fixing screws and plaques.

**Figure 4:**
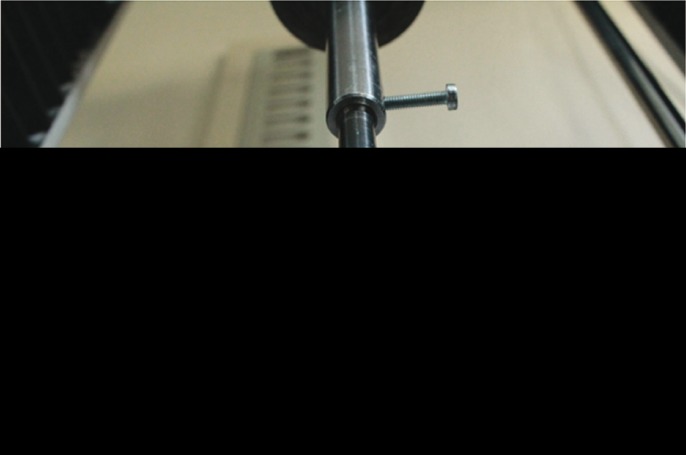
We made a fork sitting in the nasal cavity to be able to perform the pulling process.

For the measurement of the strain, these gauges were attached to 6 different anatomical sites (Fig. 2). A Following the attaching procedure, each model was mounted on a servo-hydraulic testing unit (TST 2500 mxe, ELISTA Electronic Informatics System Design Ltd) (Fig. 3). In order to mimic downfracture, pulling force was applied to prepared skull models upwardly. To be able to fulfill the pulling process, we made a fork sitting in the nasal cavity (Fig. 4). The testing unit was equipped with a 2500-kg load cell (maximum load capacity of 5000 kg), which was set to produce linear displacement at a rate of 10 mm/min. Each model was then subjected to a continuous linear tension until a plastic deformation was seen.

Strain values were analyzed with non-parametric Mann-Whitney U test by using SPSS 20 Package Program (SPSS Inc., Chicago, IL).

## Results

In the macroscopic examination of the skull model at the end of the experiment, high level pterygoid plate fracture were defined as those that occurred above the level of the Le Fort I osteotomy or near the base of the skull. Low-level pterygoid plate fractures were defined as those that occurred below the level of the Le Fort I osteotomy. In the group A, we observed low-level fractures in the horizontal direction on both the medial and lateral pterygoid plates in all skull models. However, no fracture on the pterygoid plates was observed except for the two skull models in the group B. In addition, no fractures were detected on the base of skull in both groups.

After the pterygomaxillary separation, we observed that the values of strain reduced but the statistical analyses showed that there were no significant differences (*p*>.05) between the 2 groups ( Table 1). Moreover, no statistical differences were found between the two groups in terms of applied forces ( Table 2). Besides that, a maximum tensile strain was recorded on the left medial pterygoid plate in the group A and a large compressive strain was recorded on the left skull base in the same group. In both groups, tensile strains were observed at the pterygoid plates whereas compressive strains were observed on the skull base.

**Table 1:**
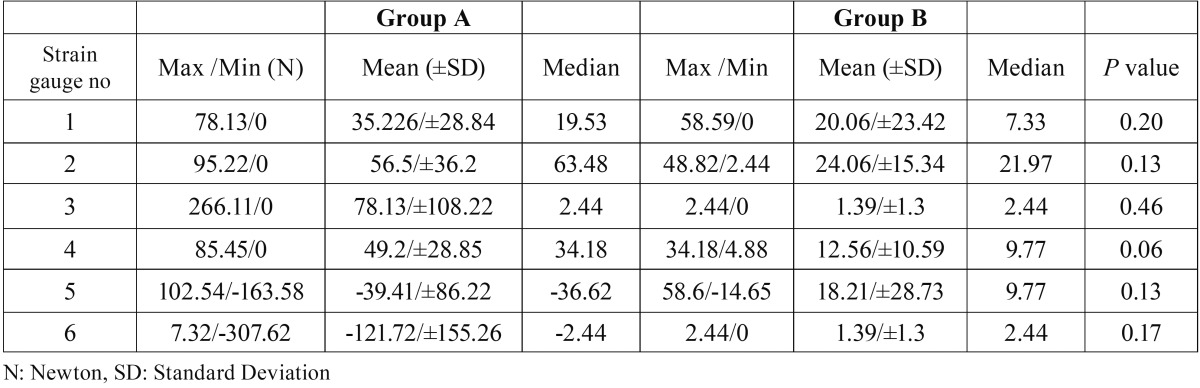
Statistical description of strain values of the two groups and *p* values.

**Table 2:**
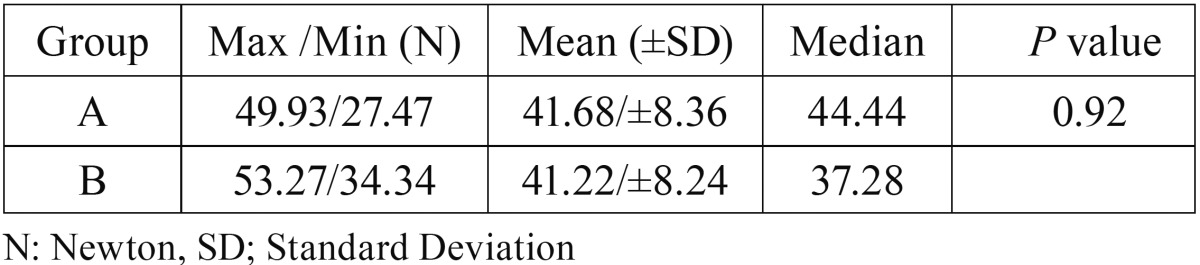
No statistically differences were found between the two groups regarding maximum forces.

## Discussion

Le fort 1 osteotomy is one of the most common methods used in correcting dentofacial deformities. It is generally known a reliable and commonly preferred procedure. Though it has a low complication rate, the researchers still work on improving the technique to avoid its permanent neurological disorders that might occur. There are few reports related to major neurological complications in Le Fort I osteotomy. Lanigan *et al.* (3) reported two cases resulting in blindness. Both of these cases were detected on early postoperative period after Le Fort I surgery. They observed that many fractures extending to the base of the skull and a bone fragment that causes direct trauma to the optic nerve on the medial part of the maxillary sinus on CT scans. The authors stated that the ophthalmic injuries appear to be primarily mediated through indirect injuries to neurovascular structures occurring from traction or compression from forces transmitted during the pterygomaxillary dysjunction using an osteotome or during the maxillary downfracture. Cruz and dos Santos (2) also reported a case of visual loss arising from the complex fractures of the pterygoid plates combined with a fracture involved the inferior and superior orbital fissures with a bone fragment extending from the superior orbital fissure to the orbital apex following Le Fort I osteotomy. The authors concluded that adverse transmission of forces via the sphenoid bone to the base of the skull during separation of the pterygomaxillary junction may explain the vascular and neuro-ophthalmic complications.

Nerve palsies were also reported in the literature. Herold and Falworth (13) reported that a pupil sparing palsy of the oculomotor nerve after a standard Le Fort I osteotomy. They thought that it was caused by ischemia of the nerve secondary to local injury by hematoma or instrumentation. Newlands *et al.* (14) stated that an ipsilateral abducens nerve palsy and partial oculomotor nerve palsy following Le Fort 1 osteotomy. They observed that a fracture reaching superior orbital fissure through the large sphenoid wing. They suggested that care should be taken when using osteotome in the pterygomaxillary fissure, particularly in those prone to untoward fractures such as older or cleft-lip and palate patients. Hanu-Cernat and Hall (1) also reported a case of late postoperative onset of abducens palsy caused by a hairline fracture of the sphenoidal sinus wall extended towards the orbit in a non-cleft, non-syndromic healthy patient. Because the aforementioned nerves extend throughout the cavernous sinus before entering into the orbital region, these nerves are also susceptible to mechanical pressure caused by bleeding (1,6,13,14).

The separation of the pterygomaxillary junction with a curved osteotome through a blind approach to the pterygomaxillary fissure is known as a standard technique of Le Fort I surgery (15). However, the necessity of separating the pterygomaxillary junction with an osteotome is still controversial (16). Some clinical and experimental studies have shown that the fractures of the pterygoid plates can occur at different levels (9,11,17,18). High level fracture of the pterygoid plates is thought to be an important factor in the occurrence of neurovascular complications (7,10). In addition, it was reported that the indirect trauma that occurs during the osteotomy may increase the possibility of injury on the anatomical structures (19). The development of the fractures of high level pterygoid plates or the forming of fine fissure fractures extend to skull base are considered to be main reasons for the occurrence of significant neurovascular morbidity (3,6). Therefore, in order to provide the mobilization of the maxilla, clinical studies are directed to pterygomaxillary separation without the use of a chisel (8,20). Our object was to evaluate the stress occurring on the anatomical areas where possible complications might develop during the downfracture for both methods.

In the literature, there are few studies investigating the mechanical properties of Le Fort I osteotomy using strain gauges (12,21,22). In these studies, the loads were applied intermittently. The data were recorded after applying each load and devices were re-adjusted to zero. However, downfracture stage is a dynamic condition that shows the continuity. During this stage, the forces affect the whole structure in an uninterrupted manner. Therefore, observing this dynamic condition can only be possible via applying the measurements also in a dynamic way. Data of the present study were recorded at 10 msec intervals during the test device moving at a constant velocity. Thus, the changes in the strains were observed from the beginning of the experiment. Maximum strain values could be measured at the moment of plastic deformation.

Materials can be exposed to two types of stresses consist of tensile and compressive stresses. These stresses are defined as the force per unit area. The main difference between tensile and compressive stress is that tensile stress results in elongation whereas compressive stress results in shortening. A material under a tensile stress returns to its original shape when the load is removed. This property of the material is known as the elasticity. However, the elastic property of a material can be observed only up to a certain value of the tensile stress, called the yield strength of the material. Thereafter, the material undergoes a permanent deformation and does not return to its original shape even if the external tensile force is completely removed. The brittle materials such as bone undergo a small amount of plastic deformation. Compressive stress is the opposite of tensile stress. When a clamping force is applied on the object, the compressive stress is formed in the region. In the present study, the tensile strains were observed at the pterygoid plates. In addition, the highest values were found in the medial pterygoid plates. Hiranuma *et al.* (12) also reported that a large strain was measured at the medial pterygoid plate in their study. Thereby, it can be considered that during both the pterygomaxillary osteotomy and downfracture the risk of fracture in the medial pterygoid plate is higher than in other regions.

There were no statistically significant differences between the two groups in terms of compressive strain values. Considering the clinical conditions, the present study shows that when Le Fort I osteotomy performed without pterygomaxillary separation, there is no significant stress on the skull base during the downfracture. However, the high compressive strains were observed at the base of skull in the non-separated group during downfracture. Additionally, even though pterygomaxillary separation reduces the strains on the pterygoid plates during downward pressure, it does not fully eliminate the risk of fracture on the pterygoid plates. Based on these findings, the results of our study support the clinical trials of Precious *et al.* (8,20). There were also no statistical differences found between the two groups regarding applied forces. It is considered that there is no need for an excessive force applied to perform downfracture in conditions that pterygomaxillary separation is not applied.

According to the results of the present study, it can be thought that the skull base fractures occurring after the Le Fort I surgery are related to the manner in which the osteotomy performed rather than the presence of osteotomy in pterygomaxillary region. Therefore, new studies that are conducted for this purpose can focus on measuring strains occurring skull base during the conventional pterygomaxillary osteotomy performed with the use of mallet. We do not know that the amount of strain in which time. Is it more during the pterygomaxillary osteotomy or during the downfracture without pterygomaxillary separation?

Although the composite models obtained via tomographic data are more fragile when compared to bone structure, we think that these models can be used largely in experimental biomechanical studies associated with orthognathic surgery. However, the verification of the present study on a cadaveric model may increase the reliability of the study. The present study conducted on a standard model with normal anatomic structure is insufficient to explain exceptional circumstances such as cleft lip and palate. When investigating the effects of the occurring forces to cranial base and other tissues during the pterygomaxillary osteotomy, development of the models that show the anatomical variations is to be point.
